# Disparities Education Strategies in the Case Comprehensive Cancer Center STEM Focused Youth Enjoy Science Program

**DOI:** 10.15695/jstem/v5i2.07

**Published:** 2022-08-31

**Authors:** Ese-Onosen Omoijuanfo, Joseph T. Williams, Kelli Qua, Jennifer Cullen, Erika Trapl, Cynthia Owusu, Damian J. Junk, Nathan A. Berger

**Affiliations:** 1 Center for Science, Health and Society, Case Western Reserve University, Cleveland OH; 2 Center for Medical Education, Case Western Reserve University, Cleveland OH; 3 Department of Population and Quantitative Health Science, Case Western Reserve University, Cleveland OH; 4 Case Comprehensive Cancer Center, Case Western Reserve University, Cleveland OH; 5 Department of Medicine, Case Western Reserve University, Cleveland OH

**Keywords:** Disparities Education, Cancer, Cancer Disparities, Healthcare Equity, Youth Enjoy Science Programs

## Abstract

The Youth Enjoy Science/Scientific Enrichment and Opportunity (YES/SEO) Program at Case Western Reserve University (CWRU) School of Medicine and the Case Comprehensive Cancer Center (Case CCC) in Cleveland, OH is an intensive Research, Science, Technology, Engineering, and Mathematics (STEM) program targeted at engaging underrepresented minority high school students to better understand and to pursue careers in cancer research and healthcare. The program’s long-range goals are to increase diversity of the cancer professional workforce to contribute to elimination of cancer health inequities. A challenging aspect of this intensive research and STEM education program is how to effectively teach about cancer health disparities and to address the importance of developing strategies for their remediation. We describe herein some innovative approaches utilized to engage students in learning about disparities and thinking about solutions. Overall, feedback from our students indicates the importance of introducing disparities education topics often and using multiple approaches, including small and large meetings as well as lecture and conversational formats. These approaches provide opportunities for frequent student engagement and concept reinforcement. Based on this experience, a series of recommendations are provided for incorporating disparities education into intensive research and STEM programs.

## INTRODUCTION

Preparing students to better understand and increase access to opportunities for careers in Science, Technology, Engineering and Mathematics (STEM) is an important national priority and numerous programs have been developed to improve mastery of STEM subjects at all levels of education across the United States (United States Dept Education 2007; National Science Foundation 2020; National Academies of Science 2021). At the same time, healthcare and biomedical professionals have noted the increased burden of diseases suffered by underserved minority populations in the U.S. and that these underserved groups are generally underrepresented in the biomedical workforce (Ford et al., 2013; Carratala and Maxwell, 2020; Palmer Kelly et al., 2021; Michaud et al., 2021). For example, in the U.S. the average age adjusted cancer mortality rate for all sites is 177.5 per 100,000 Black persons compared to 156.3 per 100,000 White persons. In Cuyahoga County, Ohio, where Cleveland and the Case Comprehensive Cancer Center are located, the average age adjusted cancer mortality rate is even worse at 201.2 per 100,000 Black individuals compared to 164.6 per 100,000 White individuals (Cuyahoga County Cancer Profile 2021). Underrepresentation of minorities in the scientific and healthcare workforce is apparent as shown by data indicating that while the U.S. population is 12.4% Black and 18.5% Hispanic (United States Census Bureau Quick Facts 2021), the national biomedical science workforce is only 2% Black and 6% Hispanic and the Oncology Physician workforce is 2.3% Black and 3% Hispanic (American Society Clinical Oncology 2015; McDowell and Heggeness, 2017; Winkfield et al., 2017; AACR 2020). The consequences of cancer disparities are most notable in that both in Cuyahoga County and across the United States, White people have higher cancer incidence rates than Black people, whereas in contrast, Black people have higher cancer mortality rates than White people.

Cancer health disparities in incidence and outcomes affect multiple organ sites and have been identified in multiple racial/ethnic minority groups including African Americans/Black, American Indian/Alaskan Natives, Asians, Native Hawaiian/Pacific islanders, Hispanic/Latinos as well as other marginalized groups including Appalachian and rural populations (Collett et al., 2022; Zavala et al., 2022). The etiology of these disparities has been attributed to multiple causes including biological and behavioral factors, structural inequities and racism, socioeconomic determinants of health, environmental factors, as well as availability and quality of healthcare services. Overcoming these disparities to achieve health equity requires commitment and action at multiple levels, including education and understanding of contributing factors (Zavala et al., 2022).

To address cancer health disparities and the underrepresentation of minorities in the cancer workforce, the U.S. National Cancer Institute (NCI) has developed a comprehensive research and training agenda to improve health equity and expand diversity in the cancer research and healthcare workforce, targeted at reducing the burden of cancer among underrepresented minorities (URMs) (National Cancer Institute 2022). An important component of this strategy is to attract and educate students at early stages of their training to better understand cancer and STEM subjects and to encourage their pursuit of careers in cancer research and care delivery (National Cancer Institute 2021). Several institutions, including our own, as well as those contributing to the Journal of STEM Outreach 2022, Special Issue on YES programs, have published descriptions of their programs to encourage and educate URM high school students and encourage their pursuit of careers in cancer research (Herek et al., 2019; Qua et al., 2020a; Lopez et al., 2021; Yanagihara et al., 2021; Collett et al., 2021; Odedina et al., 2022). These reports have generally focused on strategies for recruiting and training URM students in STEM subjects. However, only limited institutions have reported approaches to disparities education (Idoate et al., 2021; Hudson et al., 2022).

At Case Western Reserve University (CWRU) and the Case Comprehensive Cancer Center (Case CCC) in Cleveland, OH, the Youth Enjoy Science/ Scientific Enrichment Opportunity (YES/SEO) program has been developed to engage high school students to focus on cancer related STEM education and to motivate them to pursue related biomedical careers (Qua et al., 2020a). The YES component of the program funded by the NCI, recruits Cleveland area students who are URMs, while the SEO component of the program, funded by local foundations and philanthropy, has broader representation from the surrounding area. The two components, YES and SEO, are operated as a single overall program focused on immersing students in individual mentored research projects and providing them with related cancer research STEM education activities (Qua et al., 2020a). Motivating students to pursue these career opportunities is expected to contribute to their own personal development, fulfillment and success. Importantly, increasing the number of URMs in science and cancer research is expected to broaden diversity of the scientific workforce, increase scientific creativity, and expand the cancer research agenda. Moreover, since researchers and health professionals often focus their work on topics that affect individuals “like themselves”, it is expected that increasing the number of URMs in cancer research and healthcare will contribute to eliminating health disparities, and related cancer morbidity and mortality (Winkfield et al., 2017; AACR 2020; Carpten et al., 2021).

Many of the YES/SEO students and their families have already experienced different aspects of inequities, in both the quality of their educational experiences as well as in their personal encounters with the health care system. Designing approaches to educate students about disparities and discrimination, their impact on their own career development and personal health, and to engage their interest and commitment to eliminate these problems is challenging in its own right. This challenge is further amplified by the need to effectively integrate these subjects into an intensive STEM oriented research and education program. Moreover, during the course of preparing URM students to pursue professional STEM careers, it is important to provide them with strategies to recognize and overcome unique challenges, such as stereotype threat, racial microaggression, impostor phenomenon, and adverse coping strategies such as John Henryism (Museus et al., 2011; Peteet et al., 2015; Sue et al., 2007; Sue et al., 2009; Fischer, 2010; Hudson et al., 2016). Accordingly, education and open discussion of these topics is expected to help students deal with these issues and promote self-esteem (Nadal et al., 2014).

This manuscript describes the evolution of our program at CWRU and the Case CCC to educate and engage YES/SEO high school students, regarding disparities and discrimination. It further provides an assessment of student impact, and it outlines recommendations for incorporating these important subjects into curriculum design.

## METHODS

### Overall Approach.

The YES/SEO program at CWRU and the Case CCC (Qua et al., 2020a; 2021) recruits students from Cleveland, OH and surrounding area suburban high schools. Students are required to be at least 14 years of age, have a GPA of at least 3.0, to be interested in biomedical research as indicated in their answers to questions on their applications, and to have two letters of recommendation, one from a science teacher and another from a guidance counselor. Applications are reviewed by a panel of high school guidance counselors and CWRU School of Medicine faculty to select students for participation in the 2–3 month summer program. In 2021, 103 high school students participated in the Case CCC YES/SEO program. Table 1 shows this group was balanced for male and female students. Most of the students were in the 16–17-year-old group, with very limited numbers aged 14 years old. The largest participating racial/ethnic group was African Americans (42.7%), followed by Asian Americans (33.9%), Whites (16.5%) and Hispanics (2.9%). Approximately 27.2% of students came from urban high schools in the city of Cleveland, whereas 72.8% came from suburban schools, most of which are immediately adjacent to the city of Cleveland. Of these schools, 67.1% were public, 24.2% were private and 7.8% were religious. Most students in the summer program had just completed 10th or 11th grade, with smaller groups coming from 9th or 12th grades, and two students having just completed 8th grade.

At the beginning of the summer program, in a two-day orientation workshop, students were trained in laboratory safety, responsible conduct of research, principles of laboratory, clinical, and community-based investigation. During the first week of the program, each student was matched with a faculty mentor aligned with the student’s interest, as indicated in his/her application, and then deeply immersed in an individually mentor guided research project. During the summer 2021 program, 15 students conducted their summer research projects on health disparity related topics, while 88 others conducted their individually mentored research on various aspects of molecular, genetic, epidemiologic, and therapeutic aspects of cancer research. Students were engaged in these research projects, in their designated labs on a fulltime basis throughout the summer program.

The summer research activities are heavily supplemented with educational activities arranged to occur as daily seminars throughout the summer program in order to extend knowledge in STEM and related cancer research fields. These educational components (Qua et al., 2020a, b, 2021) include Lunch and Learn Research Seminars, Science in the News Workshops, Cancer Disease Specific Seminars, Focused Book Reviews, Near Peer Mentor Workshops, Writing Workshops, Career Cafés and others.

### Educational Activities.

Those that have been employed in our approach to disparities education are described below. Importantly, all educational activities, whether large or small, were conducted in mixed groups to encourage diversity of viewpoints and understanding.

Lunch and Learn Research Seminars have been delivered in person, or virtually, two times each week, during the entire program (Qua et al., 2021). These seminars are delivered by faculty members who present on various research topics in order to familiarize students with the depth and breadth of research that takes place at the Case CCC and Case SOM. Faculty are asked to discuss three components in each seminar. First, they are asked to briefly explain their academic discipline, second, its cancer relation, and third, to describe their own career trajectory and any hurdles that they had to overcome.

Cancer disease specific seminars are one-hour sessions that occur once weekly. The focus of these sessions is to convey basic understanding of pathophysiology and therapeutic approaches for different types of cancers. The seminars are conducted and delivered by MD and MD/PhD students under faculty supervision. Since the advent of COVID-19, these one-hour sessions were delivered virtually in a question-and-answer format to encourage student engagement and active learning.

Near Peer Mentor Workshops are weekly sessions that are led by MD/PhD students. These sessions occur in small groups consisting of approximately 10 students, arranged to reflect diversity, to encourage them to discuss relevant topics and issues, which focus on student wellness, resilience, stress and coping strategies. The MD/PhD students first meet with high school guidance counselors to learn best practices for working with and engaging high school students. (Qua et al., 2020b).

Focused book discussions were used as another educational technique employed by the program. At the start of the research year, each student was given a book to serve as a catalyst for open dialogue about the specific social challenges the students may encounter in their personal experiences. In choosing the book, the students’ age and academic levels were taken into account (Qua et al., 2021). Examples of books used in the program include the *Curious Incident of the Dog in the Night-Time* by Mark Haddon; *Story of a Girl* by Sarah Zarr; *We Beat the Street* by Drs. Sampson Davis, George Jenkins, and Rameck Hunt; and *One of Us is Lying* by Karen McManus. Near the end of the summer program, students participated in a review session where they analyzed the book, often discussed its relevance to their own experiences and the insights they gained regarding career development. The book review experience was led by an URM faculty member whose academic role in the School of Medicine is to foster student diversity and inclusion. This faculty member played a major role in book selection and supplemented the conversation with his own experiences. While this activity was successful in pre pandemic years, it was discontinued in 2020 and replaced by a weekly program, Science In The News, to engage students on a more frequent basis.

Science In The News workshops, held weekly, were further used to intentionally introduce disparities education and to provide students space to share their opinions of current events based on scientific understanding and to further begin developing strategies for promoting health equity. These sessions emphasized the conversational approach to learning, in which principles to be learned emerged from conversations guided by the leader, with extensive input based on student assigned readings and on student experiences (Pask, 1975; Baker et al., 2002). Students were provided with focused articles from scientific literature and news media, then asked to discuss the material based on student assigned readings and on their own experiences of how individuals interact with the healthcare system. Students were also asked to identify possible barriers that may contribute to disparities and how they might be remedied. Science In The News conversations were guided by an URM faculty member, who as noted above was focused on diversity and inclusion. In addition, faculty members with content expertise were invited to participate in discussions to guide students through interpretation of data, news, or literature and to provide more insight into specific student questions.

Topics discussed during these Science In The News workshops included “Environmental Effects on Prenatal Development and Neonatal Health Mortality; Bayer $10 Billion Settlement to Resolve the Lawsuit about Weed Killer Carcinogenesis; The Obesity Pandemic and Cancer; Concepts of Race and Racism and the Impact on Healthcare Inequity; Covid-19 Infections, Evidence and Reasons for Ethnic Differences; and a Pandemic Associated Rationale for School Openings or Remote Delivery. An interesting approach for the School Opening discussions was to have students examine issues from the viewpoint of the different stakeholders, for example, students, teachers, principals, star athletes seeking college scholarships, bus drivers, parents, public health officials, students receiving school-based food supplements, SARS-CoV-2 vaccine development and uptake, etc. (Qua et al., 2021; Junk and Berger, 2022). This approach was useful, both for analyzing situations from multiple viewpoints, and also for engaging more students in discussion. It has recently been suggested that focusing on disparities associated with COVID-19 and its burden among minority groups may lessen concerns among White U.S. residents (Skinner-Dorkeenoo et al., 2022). While we did not formally evaluate such behaviors, it appeared that students of all backgrounds were increasingly observant of mitigation strategies based on their knowledge of disease pathogenesis acquired as part of our program. In addition, many students of different backgrounds related how they used their knowledge, acquired in our discussions, to urge their family members to get vaccinated.

Students were provided with weekly Career Café presentations about career opportunities by faculty members representing different clinical and/or research careers, including public health and preventative medicine, medical oncology, surgical oncology, radiation oncology, cellular therapy, neurosurgery, developmental therapeutics, and others. Faculty members were asked to describe the scope of their professional activities, training requirements, hurdles to overcome, and aspects of professional satisfaction. To the extent possible, faculty members selected for these presentations were members of URM Groups.

Since these presentations by each faculty member occur once per year, to discuss their particular specialty, they do not represent undue burden on any individual faculty member. In fact, most faculty members enjoy these presentations to young students, and are eager to speak in this forum. It should be noted that faculty members are not directly compensated for these talks outside of their overall academic salaries, however they are provided with letters from leadership in support of their promotion and tenure applications. In addition, many faculty members use these presentations to engage students to shadow them and/or help with their research programs.

### Program Development.

In early years of the program, before 2018, health disparities and racism were not explicitly addressed. Rather the book review served as an indirect approach to address discrimination and disparities, by encouraging students to analyze and discuss obstacles encountered by book characters and strategies used to overcome them, related to their own unique experiences. In 2018, the topic of Cancer Disparities was specifically discussed in a single Lunch and Learn Seminar Session. In addition, presenters who self-identified as members of underrepresented populations shared how that identity played a role, positive or negative, in their career and professional development and discussed coping strategies that they used. Further, there was an implicit inclusion of the effects of disparities and racism that could be derived from the expert faculty members who were URMs and talked about their career development experiences. However, while many sessions were led by faculty who are URMs, there was not a prevalence of material that focused directly on disparities in healthcare.

In 2020 and 2021, more deliberate efforts were made to incorporate disparities education across multiple program venues. In addition to a specific Lunch and Learn Seminar, discussions of disparities were included in multiple disease specific seminars focused on breast cancer, myeloma, endometrial cancer, and others.

Near Peer Mentor Groups are also a method where students were encouraged to discuss disparities in a smaller, more personal environment. As related by students in focus groups, this latter activity may have allowed for the most organic incorporation of disparity education, thus smaller groups allowed discussions to be shaped by students’ interests and experiences.

Career Cafés took yet another approach when incorporating education about disparities especially when sessions were led by URMs who discussed their own experiences and the direct impact of their identity and background.

In 2020, the omnipresent and inescapable news media focus on the COVID-19 pandemic, the recognition of both cancer and race/ethnicity, as factors for the SARS-CoV-2 virus infection and severity of illness, precipitated extensive discussions in the Science In The News workshops. These conversations focused not only on school closings, as noted above, but also on potential mechanisms for disparities, their relation to structural racism and approaches for their control. A similar situation, leading to extensive Science In The News discussions, occurred in 2021, surrounding race/ethnicity issues, vaccine advocacy, uptake, resistance, and hesitancy. These discussions routinely addressed minority mistrust of the medical research establishment based on historical examples including the Tuskegee Study and the Henrietta Lacks Story (Bajaj and Stanford, 2021). For these meetings, students were asked to identify possible factors that might contribute to disparities in their own environment and then students were explicitly asked to identify possible factors that influenced how individuals access and resource health services or healthcare. On a more personal level, students were encouraged to discuss their own experiences with the medical community, as well as with the COVID-19 pandemic. These sessions were highly interactive, undoubtedly due to the students’ personal knowledge of individuals and family members that contracted COVID-19, and because of the constant bombardment from the news media. These weekly conversations were most engaging and student participation was very high.

The fact that these conversations were led by a URM faculty member almost certainly contributed to student relatability and comfort while discussing these uncomfortable topics. While Science in the News discussions were among the liveliest of the summer program, they remained very respectful, even as students expressed a wide and diverse range of opinions.

As already noted, the major focus of the YES/SEO Program is individually mentored research immersion. Among 103 individual research projects, most of which were wet lab based, at least 15 students conducted disparities related projects as part of their Research Immersion, with results presented at the Capstone Program. At the end of the summer program, all students participated in the Capstone Research poster presentation. Each student prepared an abstract, which was published in a program booklet, then each student formally presented a summary of their research to an audience of peers, faculty, laboratory personnel, family, friends and community leaders. In previous years, the Capstone Poster presentation was held in-person. In 2020 and 2021, the presentations were virtual (CWRU, 2021).

A new program entitled Community Engagement Research Program (CERP), introduced after the completion of the 2021 Summer Program, focused on engaging students to improve community health, often employing efforts to identify, reduce, and prevent disparities. This program engaged students who have already participated in the summer research program, for a new 30 week-long program, consisting of three to five hours per week, supported by the R25 YES award, where students learned the principles of community-based participatory research. They subsequently interacted with community members, to apply principles of Community Engagement in Science, in order to identify how biology, epidemiology and population health support cancer prevention and control. They were further provided with opportunities to recognize historical and ethical considerations for conducting human research, and to utilize qualitative and quantitative methods for problem-based research study and apply translational strategies to support the uptake of community engaged research. In its first year, the program attracted and sustained participation by 12 students during their full-time high school studies. Other than these students that participate in this unique, new CERP Program, all students return to their regular school studies, while variable numbers continue to interact, on an individual basis with their research project labs.

Every spring the Case CCC hosts a two-day regional Cancer Disparities Symposium featuring prominent keynote speakers along with poster and platform presentations. In recent years, a highlight of the meetings has been a special session, where after keynote presentations, YES/SEO and selected high school students are provided an opportunity to meet privately in a question-and-answer session with each speaker. In 2021, students met with Dr. Otis Brawley, Bloomberg Distinguished Professor, Johns Hopkins University and past Chief Medical Officer of the American Cancer Society; while in 2022 they met with Dr. Robert Winn, Professor of Medicine and Director Massey Cancer Center, Virginia Commonwealth University: and Dr. Lori Pierce, Professor Radiation Oncology, University of Michigan and past President American Society of Clinical Oncology. These meetings with minority group members who had achieved leadership status in American Medicine, were described as “awesome” by both students and speakers who were clearly inspired by each other.

### Program Assessment - Data Analysis.

All program evaluation activities were reviewed by the CWRU Institutional Review Board (IRB) (STUDY #20190701). YES/SEO students provide consent for the inclusion of the responses for publication. All evaluation activities were voluntary and all replies were anonymous.

A week prior to 2021 YES/SEO program, all participating students were invited to complete a two-part electronic pre-program survey. The first component of the survey consisted of 16 overarching questions centered on student interest, knowledge of cancer related topics, disparities, wellness, and community resources. The survey used closed-ended questions including multiple-choice, ranking, and matrix-style Likert scale questions. Open-ended questions were included for additional elaboration.

During the final week of 2021, all YES/SEO program students were invited to complete an extended 48-item post-program survey. The post-program evaluation survey contained the same questions as the pre-program evaluation survey but also included additional information on program satisfaction, experience with Near Peer Mentors (a core component of the YES/SEO program) and future plans, including interest in certain degree programs and universities.

Two weeks after completion of the program, all students were sent a seven-item survey unique to the 2021 YES/SEO program. It asked students a series of questions related to the YES/SEO disparities curriculum. One of the seven items was an extended matrix-question asking students to rate their level of agreement on a variety of disparities related topics including knowledge, awareness, and effectiveness of the related YES/SEO curriculum.

All program evaluation surveys were sent via Qualtrics to student email addresses using unique links. Students received up to three reminders to complete surveys.

Quantitative data are reported using descriptive statistics. Qualitative data were analyzed using open coding for general themes. Two members of the research team (EO, KQ) independently read qualitative comments line by line twice to identify a list of general themes, sentiments, and patterns presented by the data. The list of possible open codes was then reviewed by two members of the research team (EO, KQ) and formalized into a set of codes with descriptions to be used for analysis. The same two members of the research team re-read and assigned codes to the data, met, compared, and discussed discrepancies, and finally recoded data to reach consensus.

## RESULTS

As noted in methods, at the beginning of the summer, all students were deeply immersed in individually mentored research projects on topics related to student interests indicated in their applications. Of the 103 students participating in these individually mentored research projects, 15 conducted their research on disparities related projects (Table 2). These projects covered a broad range of approaches across the spectrum from molecular genetics, to immunologic, psychosocial, epidemiologic, and community-based investigation. One of the students was recognized as co-author on an article published in the peer reviewed scientific literature (Wang et al., 2022). Of the 15 students whose summer research projects were related to disparities, two were motivated to participate in the newly developed Community Engagement Research Program (CERP). The other students who signed up for the extended CERP were motivated to do so by involvement with the overall YES/SEO educational program.

**Figure 1.**
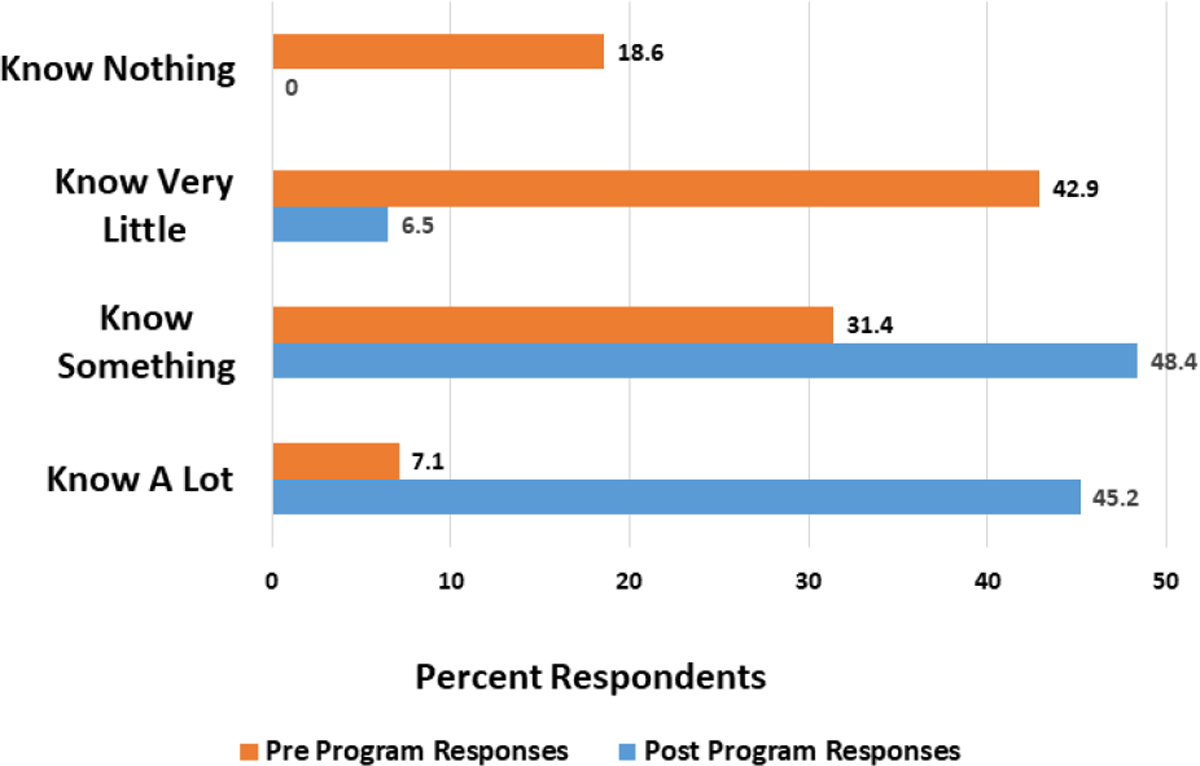
Knowledge of Cancer Disparities Among High School Students Participating in 2021 Case CCC YES/SEO Program.

Figure 1 shows that in a pre-program survey, 18.6% of students reported that they knew nothing about healthcare disparities, 42.9% reported they knew very little, 31.5% knew something, and 7.1% said they knew a lot. In a post program survey 45.2% of students reported that they knew a lot about healthcare disparities, 48.4% knew something, while only 5.5% reported they knew very little.

As reported in Table 3 students indicated that multiple venues in the YES/SEO Educational Programs all contributed to their increased knowledge about inequities and disparities. The Science in the News workshops were identified by 87% as the program format where most learned about disparities and inequities. Further 68.4% and 65.8% reported that the Lunch and Learn Seminars and the Career Cafés were effective programs for educating them about disparities and inequities. 46% of program students also identified the Near Peer Mentoring Program as having contributed to their education about disparities and inequities. The book discussion component was not evaluated in the survey since it was not included in the 2021 program, nor was the Community Engagement Research Program, since it was initiated after the survey had already been completed. These results indicate that the multiple venues approach was effective in promoting students to learn about disparities.

As shown in Table 4, a large majority of students (84.6%) reported that disparities were topics of interest. 64.1% of students indicated they had conversations about disparities and inequities in venues other than the YES/SEO program, while 35.4% of students indicated that they did not discuss healthcare disparities or inequities in their spare time. 79.5% of students said they were inspired by the YES/SEO program to have more conversations about disparities and inequities. By the end of the summer program, 94.8% of students indicated that they learned a lot about healthcare disparities and equity, while 93% reported that the YES/SEO program helped them to better understand these subjects. Despite the students’ overall increased interest and knowledge, 12.7% of students reported they struggled to understand why disparities were discussed as part of STEM education. As noted above, these surveys were conducted anonymously so respondents’ ethnicities were not available. Nonetheless, the large majority of students who indicated they learned a lot about healthcare disparities and to better understand disparities, demonstrates an important impact on all groups of students.

In response to the open-ended question “Why do you think it’s important to have more people thinking about disease that affects minorities?”, four major themes emerged: 1. The importance of access to healthcare, 2. Collective thinking, 3. Equity, and 4. Increasing awareness of health disparities.

Nineteen % of comments indicated students’ awareness of increased health care needs and/or issues with access to health care, related to demographic and socio-economic factors. Students noted physical and environmental hindrances, such as location of services and lack of information for patients. For example, one student commented that,

Because minorities are so poorly represented in healthcare and in the media, so they’re not given the same information that is readily available to white people, or the majority. Due to lack of information, more people in minority populations are dying from disease that could have been treated and even cured if they were of a different race.

Approximately 38% of comments emphasized the importance for the next generation to think collectively about disparities and the importance of considering our community to help us progress towards addressing disparities. One student mentioned that, “Having discussions [about disparities] can drive people to put more effort into attempting to alleviate the risks of diseases and work towards a more advanced and safe society.”

Regarding the third theme of Equity, 40% of comments mentioned issues in healthcare inequity. Students were very aware of issues in both healthcare funding related to disparities, issues with access to quality care, socio-economic factors and mistrust. When reflecting on the importance and inequity of health care disparities, one student commented, “It allows us to consider the diseases that not only impact us, but also the ones that impact others. We will then be able to create a more equitable health care system, if we can reach diseases that affect everyone.”

Thirty-four % of comments addressed the need for healthcare professionals to be more aware of disparities. One student reflected, “It is important to have more people thinking about diseases that affect minorities, because it allows us to start making steps towards improving said disparities. If everyone remains ignorant of these prevalent problems, no improvements will ever be made.” Students believe that conversations should be held widely across all stakeholders to increase the likelihood that policies and innovation can be developed to decrease healthcare disparities.

## DISCUSSION

### High School Student Interest in Disparities Education.

A central theme that arose from the students’ responses to the survey’s open-ended questions was the sentiment that knowledge could lead to real action and change. This sentiment was highlighted through student comments, such as “I think it is important because people can only help to change situations that they’re actually educated in.” Students demonstrated immense insight into the power of knowledge to effect change in policy and action. One student remarked that, “if the disparities are not acknowledged they cannot be addressed” and reflected that, “the lack of knowledge leads to a continuation of the problem.” Student interest was further reflected by the 84.6% who reported that they were interested in having more conversations about disparities and inequities. Many of these students are confronted with the reality that as individuals of minority backgrounds, functioning within extremely diverse communities, the topic of healthcare disparities is directly related to their personal experiences and the experience of others within their community. Thus, even at young ages, this awareness is translated into a desire to learn more and the foresight that the knowledge itself is valuable. This quest for knowledge, makes educational components as conducted as part of the YES/SEO Program invaluable and powerful tools for providing knowledge to the high school students to ultimately contribute to the solutions and to reduce the burden of cancer in URM groups.

### Challenges to Education About Disparities.

Based on our experience, particularly in group discussions, an important challenge in incorporating educational materials that directly address disparities in healthcare is that often this topic is very sensitive, and uncomfortable. Moreover, it is hard to fully address an important issue such as disparities in healthcare in a single 90-minute session, which we attempted to do in early years. While the 2021 YES/SEO program did not provide a fully comprehensive education on disparities, it had multifaceted presentations and discussions of disparities through different ongoing components and different venues. This allowed for an immersive, comprehensive, and ongoing promotion of the issues which helped establish their importance, present accumulated knowledge, and reinforce the importance of acknowledging how complex the issues truly are. Another challenge to education on these topics is the age level of students in the program, 14–18 years old. Thus, it is important to construct educationally effective conversations about these topics, within awareness of the age and academic level of the participating students. While it was clear that interest was not an issue when initiating these lectures and conversations, it would be a disservice to students to present elements of this issue that are not age appropriate. Students showed considerable interest in the material of their own accord. Participation, attention, and engagement with the material was generally not an issue. The student interest was both self-generated and as a result of the specific form of programming. As in many educational settings, a majority of students engaged with the material and participated. Nonetheless, a minority of students participated less and needed to be encouraged to engage. The latter was accomplished by multiple approaches including asking students to relate opinions based on personal experiences, and/or assuming the roles of designated stakeholders as described above in the discussions on school closings. Nonetheless, it remains important to explore what methods can be incorporated to more fully engage all students so that every student can derive the full benefit of the educational programs.

### Recommendations for Incorporating Disparities Education into Curriculum.

Overall, it is clearly critical as a society that we address healthcare disparities in order to achieve health equity. A key challenge in this regard is how to most effectively educate about these issues among students focused on STEM based subjects and also, how to motivate future generations of all ethnicities, to not accept the situations that perpetuate disparities in our society. As with education strategies in general, our observations indicate the importance of utilizing multiple and diverse approaches to disparities education. Toward these ends, our experience suggests that for incorporating disparity education into a curriculum, it is important to use a multifaceted and frequently reinforcing approach, given the broad and complex nature of healthcare disparities. This is necessary so that students, especially those to whom the issue may be new, have the opportunity to acquire and reinforce their knowledge on the issues. Consequently, incorporating educational components that use different approaches and venues creates an immersive learning experience. For example, incorporating disparities education in Lunch and Learn Seminars, which are more lecture-based, along with workshops such as Science In The News, which are more dialogue-based, and Near Peer Mentor sessions, which are small group discussions, allow the issues to be approached from many different perspectives creating a rich learning experience for the students.

The lessons learned from our experience with YES/SEO high school students are summarized below for others planning to provide high school students with opportunities to learn about healthcare disparities and inequities and to begin to contemplate their remediation.

- Most students are aware of disparities in healthcare and inequities in the healthcare professions. Awareness is most prevalent among members of underrepresented minority groups.
- Students are eager to learn more about healthcare disparities and acknowledged that knowledge was the beginning of fixing the problems.
- Recurrent discussions of healthcare disparities using different venues effectively reinforces student knowledge and engagement in this subject.
- The use of multiple opportunities and different venues to present and review issues of disparities was important in providing students with enough comfort to freely discuss related issues
- Once the topic of disparities was introduced into the conversation, students became increasingly comfortable to participate in these discussions in both large formats (Science In The News Workshops) and small groups (Near Peer Mentor Meetings).
- Conversations led by underrepresented minorities were most successful in engaging student interest and participation in discussions of disparities.
- Discussions were most effective in engaging students when they could personally relate to the issues.
- Assigning students to research laboratories and/or programs that directly address disparity related issues is an important beginning to engage their interest in solving these problems, stemming from the molecular to the community approach.
- Our newly developed nine month long, Community Engagement Research Program, provides a unique opportunity for students who wish to dive deeply into this subject, to better understand disparities and actually engage in their remediation.

## Figures and Tables

**Table 1.** Demographic Characteristics of Students Participating in the Case Comprehensive Cancer Center 2021 YES/SEO Program.

Total	N = 103	(100%)

**Gender**		
Female	55	(53.3%)
Male	46	(44.6%)
Non-Binary	2	(1.9%)
**Age (Years)**		
14	6	(5.8%)
15	18	(17.4%)
16	30	(29.1%)
17	33	(32.0%)
18	16	(15.5%)
**Ethnicity**		
African American	44	(42.7%)
Asian	35	(33.9%)
Hispanic	3	(2.9%)
White	17	(16.5%)
**Schools Attended**		
Urban	28	(27.2%)
Suburban	75	(72.8%)
Public	70	(67.9%)
Religious	8	(7.8%)
**School Grade**		
Eighth	2	(1.9%)
Ninth	16	(15.5%)
Tenth	29	(28.1%)
Eleventh	39	(37.8%)
Twelfth	17	(16.5%)

**Table 2.** Youth Engaged in Science Cancer Disparity Related Research Projects.

1.	Risk of MIZ-1 in Triple Negative Breast Cancer and Cell Proliferation and Disparities
2.	Triple Negative Breast Cancer in African American Women
3.	Young Adult Substitution of Flavored Cigarillos with Menthol Cigarettes
4.	How Electronic Cigarettes and other Tobacco Products Affect Your Lungs
5.	Flavored Tobacco Product Use Among the Younger Populations
6.	Effects of Targeted Marketing on Tobacco Use in Youth Minority Populations
7.	Environmental Risk Factors of Lung Cancer in Ohio
8.	African American Male Screening Results Show Greater Risk for Prostate Cancer at a Younger Age
9.	Prostate Cancer Screening Guidelines Incorporating Biologic and Sociological Factors Affecting African American Men with Prostate Cancer
10.	African American Screening Guidelines and Reasons for Different Guidelines
11.	County Based Trends of Obesity with Cancer
12.	Resiliency in African Americans and the Impact of Potentially Traumatic Events
13.	COVID-19 and Damage to the Lungs
14.	Risk, Incidence, and Disparities of COVID-19 Breakthrough Infection Among Patients with Multiple Myeloma
15.	Family Income and Its Impact on Family Resilience

**Table 3.** Case CCC High School Student Post Program Survey of YES/SEO Components Where They Learned About Healthcare Disparities and Equities.

Science In The News	87%
Near Peer Mentor Meetings	46%
Lunch and Learn Seminars	68%
Career Cafés	65%

Total greater than 100% since students were asked to identify all components where they learned about healthcare disparities.

**Table 4.** Case CCC YES/SEO High School Student Post Program Survey of Knowledge and Interest in Healthcare Disparities and Equities (HCD&E).

HCD&E is an interesting topic	84.0%
We discussed HCD&E in school	30.7%
I generally do not discuss HCD&E with family and friends	35.4%
I commonly discuss HCD&E with family and friends	64.1%
I struggle to understand why HCD&E discussions are part of STEM Education	12.7%
As a result of YES/SEO I will discuss HCD&E with friends and family	79.5%
I learned a lot about HCD&E in YES/SEO	94.8%
I better understand HCD&E as a result of YES/SEO	93.0%
Is it important to hear from mentors who belong to your racial, SES, group in the field you are pursuing	84.0%

HCD&E = Healthcare Disparities and Equity

## ABBREVIATIONS

Case CCC: Case Comprehensive Cancer Center
CERP: Community Engagement Research Program
CWRU: Case Western Reserve University
IRB: Institutional Review Board
NCI: National Cancer Institute
SEO: Scientific Enrichment and Opportunity
STEM: Science, Technology, Engineering, and Mathematics
URMs: Underrepresented Minorities
YES: Youth Enjoy Science
